# ADHD and Methylphenidate Use in Prepubertal Children and BMI and Height at Adulthood

**DOI:** 10.1001/jamanetworkopen.2025.52019

**Published:** 2026-01-05

**Authors:** Jihun Song, Sun Jae Park, Jiwon Yu, Jina Chung, Seogsong Jeong, Sang Min Park

**Affiliations:** 1 Department of Biomedical Informatics, Korea University College of Medicine, Seoul, Republic of Korea; 2 Biomedical Research Center, Korea University Guro Hospital, Seoul, Republic of Korea; 3 Department of Biomedical Sciences, Seoul National University Graduate School, Seoul, Republic of Korea; 4 Department of Family Medicine, Seoul National University Hospital, Seoul, Republic of Korea

## Abstract

**Importance:**

As attention-deficit/hyperactivity disorder (ADHD) is commonly diagnosed in childhood, methylphenidate (MPH) is the most widely prescribed treatment. Although effective for symptom management, concerns remain about an association with growth and body composition.

**Objective:**

To investigate the associations of the prevalence of ADHD and MPH use in childhood with body mass index (BMI) and height at adulthood (aged 20 to 25 years).

**Design, Setting, and Participants:**

In this nationwide retrospective cohort study, data of children aged 6 to 11 years and adolescents aged 12 to 19 years with newly diagnosed ADHD from the Korean National Health Insurance Service were included between January 2008 and December 2013. Exact matched controls of children and adolescents without ADHD were also included. Their cumulative medication exposure over a 4-year period was assessed, and their height and weight during adulthood were subsequently evaluated from January 2018 to December 2022. Data extraction and statistical analyses were conducted from November 2024 to May 2025.

**Exposures:**

Diagnosis with ADHD and MPH prescriptions (cumulative days of MPH use, <1 year or 1 to 4 years, within 4 years after the diagnosis).

**Main Outcomes and Measures:**

The primary outcomes were BMI (calculated as weight in kilograms divided by height in meters squared) and height (in centimeters) at adulthood, assessed as continuous variables (crude mean [SD] and adjusted mean [95% CI]) and binary outcomes (adjusted odds ratio [AOR] [95% CI]). Measures for being overweight and obese were a BMI of 25 or more for males and 23 or more for females; measures for having short stature were a mean (SD) less than 174.4 [5.5] cm for males and less than 161.8 [5.3] cm for females.

**Results:**

In this cohort study of 34 850 youths (n = 12 866 prepubertal children aged 6-11 years; mean [SD] age, 9.3 [1.4] years; 9329 males [72.5%]; and n = 21 984 adolescents aged 12-19 years; mean [SD] age, 14.5 [1.8] years; 14 633 males [66.6%]), prepubertal children with ADHD compared with those without ADHD had a higher adjusted mean BMI (24.3 [95% CI, 24.2-24.4 vs 23.3 [95% CI, 23.2-23.4]; *P* < .001) but not a significantly shorter height (167.8 cm [95% CI, 167.7-167.9 cm] vs 167.9 cm [95% CI, 167.8-168.0 cm]; *P* = .10) at adulthood. Those with ADHD who received MPH therapy compared with participants without ADHD had greater odds of a BMI classified as overweight and obese (AOR, 1.60 [95% CI, 1.51-1.71]; *P* < .001) and short stature (AOR, 1.08 [95% CI, 1.02-1.15]; *P* = .01) at adulthood.

**Conclusions and Relevance:**

In this cohort study, patients with ADHD, particularly those treated with MPH, had a higher BMI and shorter height at adulthood than individuals without ADHD. Although the observed height difference was clinically small in both sexes and age groups, the findings suggest that long-term MPH exposure may be associated with growth and body composition, highlighting the need for regular monitoring of growth.

## Key Points

**Question:**

Is the long-term prevalence of attention-deficit/hyperactivity disorder (ADHD) and methylphenidate (MPH) use in children associated with body mass index (BMI) and height at adulthood?

**Findings:**

In this cohort study of 12 866 children aged 6 to 11 years, those with ADHD had a higher BMI and slightly shorter height than controls without ADHD. These associations were stronger among those receiving MPH therapy, particularly with long-term exposure.

**Meaning:**

The findings of this study suggest that children with ADHD, especially those receiving MPH therapy, may face greater risks of obesity and modest height reduction, highlighting the importance of continuous growth monitoring.

## Teaser

This cohort study investigates the associations of the prevalence of attention-deficit/hyperactivity disorder (ADHD) and methylphenidate use among prepubescent children and adolescents with body mass index (BMI) and height at adulthood.

## Introduction

Attention deficit/hyperactivity disorder (ADHD) is a prevalent neurodevelopmental disorder in children and adolescents.^1,2^ As a long-term management strategy for ADHD, methylphenidate (MPH) has been widely prescribed as a psychostimulant and first-line treatment due to good efficacy for alleviation of ADHD symptoms.^3,4^ However, MPH use is increasingly recognized as being associated with long-term health outcomes, including sleep disturbances,^5,6^ the onset of mood disorders,^7,8^ and increased risk of physical comorbidities.^9,10^ As one of the ongoing concerns on the adverse effects of ADHD and MPH use, an issue about the potential impacts on children’s obesity and physical growth, particularly height at adulthood, has been raised.^11^ Although previous clinical trials and observational and meta-analysis studies have suggested a possibility and a biological mechanism of disrupted growth,^12,13^ the effects of ADHD prevalence and MPH use remains unclear. Children with ADHD may be at increased risk for unhealthy lifestyle behaviors, such as irregular eating patterns,^14^ low levels of physical activity,^15^ and poor sleep quality,^16^ which may contribute to obesity and shorter height than individuals without ADHD. As MPH is well known to suppress short-term appetite,^17^ skipping meals and overeating due to appetite rebound may contribute to being overweight. Additionally, because the growth hormone is mainly released during deep sleep, chronic sleep deprivation can disrupt the hormone release and slow growth velocity, thereby interfering with optimal height development. However, an association of ADHD and MPH use with overweight and shorter height is not well-established.

Herein, we conducted a large-scale and retrospective cohort study using national health examinations and records on diagnosed disease and drug prescription in the Republic of Korea. We aimed to assess whether patients with ADHD and MPH use exhibit differences in body mass index (BMI) and height at the time they reach adulthood compared with matched controls without ADHD. Our study may offer robust evidence to inform the potential long-term physical consequences of ADHD and MPH use for clinicians, patients, and caregivers.

## Methods

### Data Source

This nationwide cohort study was approved by the institutional review board of Seoul National University Hospital and was conducted in accordance with the Declaration of Helsinki.^18^ Informed consent was waived because it did not constitute human participants research and used deidentified data. Data were obtained from the National Health Insurance Service (NHIS), which covers nearly all essential and supplementary medical services, including national health examinations, for over 97% of the Korean population.^19^ MPH use was collected based on prescriptions after the diagnosis of ADHD, and outcomes were captured only among those who completed health examinations (eFigure 1 in Supplement 1). The insurance data are managed by the NHIS (anonymized and deidentified) after they are electronically transmitted to, verified by, and stored in the NHIS database system. We examined the association between exposures, which were operationally defined and collected from insurance claims data for medication prescriptions and disease diagnoses, and outcomes, measured through the national health examination that is provided free of charge to adults (aged ≥20 years).^20^ The health examinations were performed in certified medical institutions by trained medical staff using standardized procedures. This study was designed as a retrospective cohort analysis following the Strengthening the Reporting of Observational Studies in Epidemiology (STROBE) reporting guideline.

### Study Population: Patients With ADHD and MPH Use

The study population included prepubertal children aged 6 to 11 years (main cohort) and adolescents aged 12 to 19 years at the time of enrollment. Patients newly diagnosed with ADHD (from January 2008 to December 2013) were identified using *International Statistical Classification of Diseases and Related Health Problems, Tenth Revision* codes (F90.0 and F98.8).^21^ To avoid potential miscoding, an ADHD diagnosis was confirmed in individuals who had at least 2 outpatient visits carrying these diagnostic codes. Each patient’s index date was defined as the date of the initial ADHD diagnosis. To minimize potential bias from prior treatment, 6779 patients with any history of ADHD medication use before the index date were excluded. The remaining patients were those who underwent at least 1 national health examination between January 2018 and December 2022 after reaching early adulthood (aged 20 to 25 years). Then, the cumulative days of MPH use were calculated over a 4-year window following the index date. Each patient with ADHD was exactly matched 1:1 by age, sex, and income level to a control without ADHD. The index date for each control was assigned the same calendar date as the index date of their matched patient with ADHD, ensuring synchronized observation windows. The final analytic cohort is presented in eFigure 2 in Supplement 1. When MPH use was identified through prescription claims in the NHIS database (drug code 1932), among those with ADHD, patients who received MPH prescriptions were further categorized according to their cumulative MPH exposure during the 4-year period following the ADHD diagnosis: less than 365 days or 365 to less than 1460 days. Additionally, the average dose of prescribed MPH was calculated as the ratio of the total prescribed dose to the cumulative prescription days, using the dosage information recorded at the time of each prescription (eFigure 3 in Supplement 1). Use of additional psychotropic medications, such as atomoxetine, clonidine, and antidepressant drugs, was also collected.

### BMI (Overweight and Obesity) and Height (Short Stature) at Adulthood Outcomes

To evaluate obesity and height at adulthood, the primary outcomes, including BMI (calculated as weight in kilograms divided by height in meters squared) and height (in centimeters), were collected from the available national health examination. Obesity and stature were evaluated using both continuous variables (crude mean [SD] and adjusted mean [95% CI] of BMI and height) and binary outcomes: overweight and obesity (a BMI of ≥25 for males and ≥23 for females), severe obesity (a BMI of ≥30), and short stature (mean [SD] height <174.4 [5.5] cm for males and <161.8 [5.3] cm for females), based on the criteria of the World Health Organization and the Korean Society for the Study of Obesity and the Korean Agency for Technology and Standards (2021 standards).

### Statistical Analysis

All data were collected, analyzed, and visualized using SAS, version 9.4 (SAS Institute Inc) and Prism software, version 8 (GraphPad) between November 2024 and May 2025. Statistical significance was determined using 2-tailed *P* < .05.

As continuous outcomes (BMI and height) were analyzed using multivariable linear regression models, results were reported as adjusted means with 95% CIs. Binary outcomes (overweight, severe obesity, and short stature) were evaluated using multivariable logistic regression models with adjusted odds ratios (AORs) and 95% CIs. To minimize confounding bias, the multivariable models were adjusted for potential covariates, including age at cohort entry (continuous [years]), sex (categorical [male or female]), household income level (categorical [quartiles]), Charlson Comorbidity Index (continuous), use of other ADHD medications (categorical [used or not used]), and the duration between initial diagnosis and the final health checkup (follow-up duration, continuous [years]). In the models, we measured clinical importance, using the proportion of explained variance (PES) as an indicator of effect size. PES values closer to 1 indicate a stronger explanatory contribution of the exposure, whereas values approaching 0 represent minimal explanatory contribution. We interpreted PES using standardized quantitative thresholds (PES <.01 was considered trivial, and ≥.10 was considered large). To assess the association with growth, the primary analysis was conducted among children aged 6 to 11 years (diagnosed at the prepubertal period), followed by the same analysis applied to adolescents aged 12 to 19 years (diagnosed during the growth period). Interaction outcomes were explored through stratified analyses by sex and age group at the national health examination (aged 20-22 years or aged 23-25 years).

## Results

### Baseline Characteristics

Among 36 345 eligible individuals, the study population included 34 850 patients, of whom 12 866 (36.9%) were children with ADHD (mean [SD] age, 9.3 [1.4] years; 3537 females [27.5%] and 9329 males [72.5%]). Of these children, 6816 (53.0%) had received MPH, while 6050 (47.0%) had not (eTable 1 in Supplement 1). These children were matched 1:1 with 12 866 children without ADHD. The mean (SD) age at the final health checkup was 21.8 (1.5) years. The sex ratio was comparable across groups in both children with ADHD and without ADHD. Among the 21 984 adolescents, the mean (SD) age was 14.5 (1.8) years, and the number of males was approximately twice that of females (7351 females [33.4%] and 14 633 males [66.6%]) (eTable 2 in Supplement 1).

### Risk of Overweight and Obesity With BMI and Short Stature With Height at Adulthood

Children with ADHD had a significantly higher BMI compared with those without ADHD (24.3 [95% CI, 24.2-24.4] vs 23.3 [95% CI, 23.2-23.4]; *P* < .001; PES ≤.01) as shown in the Figure and eTable 3 in Supplement 1. Among males, the adjusted mean BMI was 25.2 (95% CI, 25.1-25.3) in the group with ADHD and 24.3 (95% CI, 24.2-24.4) in the group without ADHD. Among male patients with ADHD, those who received MPH treatment had an even higher adjusted mean BMI (25.4 [95% CI, 25.3-25.6]), and a duration-response association was observed: those treated for more than 365 days (1-4 years of use) had the highest adjusted mean BMI (26.0 [95% CI, 25.7-26.3]). In contrast to BMI, the difference in height (−0.1 cm) was not statistically significant in children with ADHD (167.8 cm [95% CI, 167.7-167.9 cm] vs 167.9 cm [95% CI, 167.8-168.0 cm]; *P* = .10; PES < .001); there was a statistically significant reduction in height among children with MPH use (167.6 cm [95% CI, 167.5-167.8 cm; *P* = .01). Among females, the adjusted mean of height in the group without ADHD was 161.6 cm (95% CI, 161.5-161.8 cm), while it was 161.4 cm (95% CI, 161.2-161.6 cm) for those with ADHD and 161.2 cm (95% CI, 160.9-161.4 cm) for all females treated with MPH. Particularly, the adjusted mean of height for females treated with MPH and 365 days to less than 1460 days of use was less than 0.6 cm (161.0 cm [95% CI, 160.4-161.5 cm]) compared with females without ADHD. Even in the subgroup stratified by obesity, children treated with MPH had a lower adjusted mean of height compared with children without ADHD (eTable 4 in Supplement 1). Similar outcomes for crude and adjusted means were observed when the analysis was extended to adolescents with ADHD and MPH (eTable 5 in Supplement 1). Scatterplot analyses show a weak positive correlation between cumulative MPH use and BMI (Pearson correlation coefficients: *r* = 0.065) and a weak negative correlation with height (*r* = −0.018) in children (eFigure 4 in Supplement 1). In both males and females, an increase in total dose of MPH was significantly associated with an increase in BMI (eFigure 5 in Supplement 1).

**Figure.**
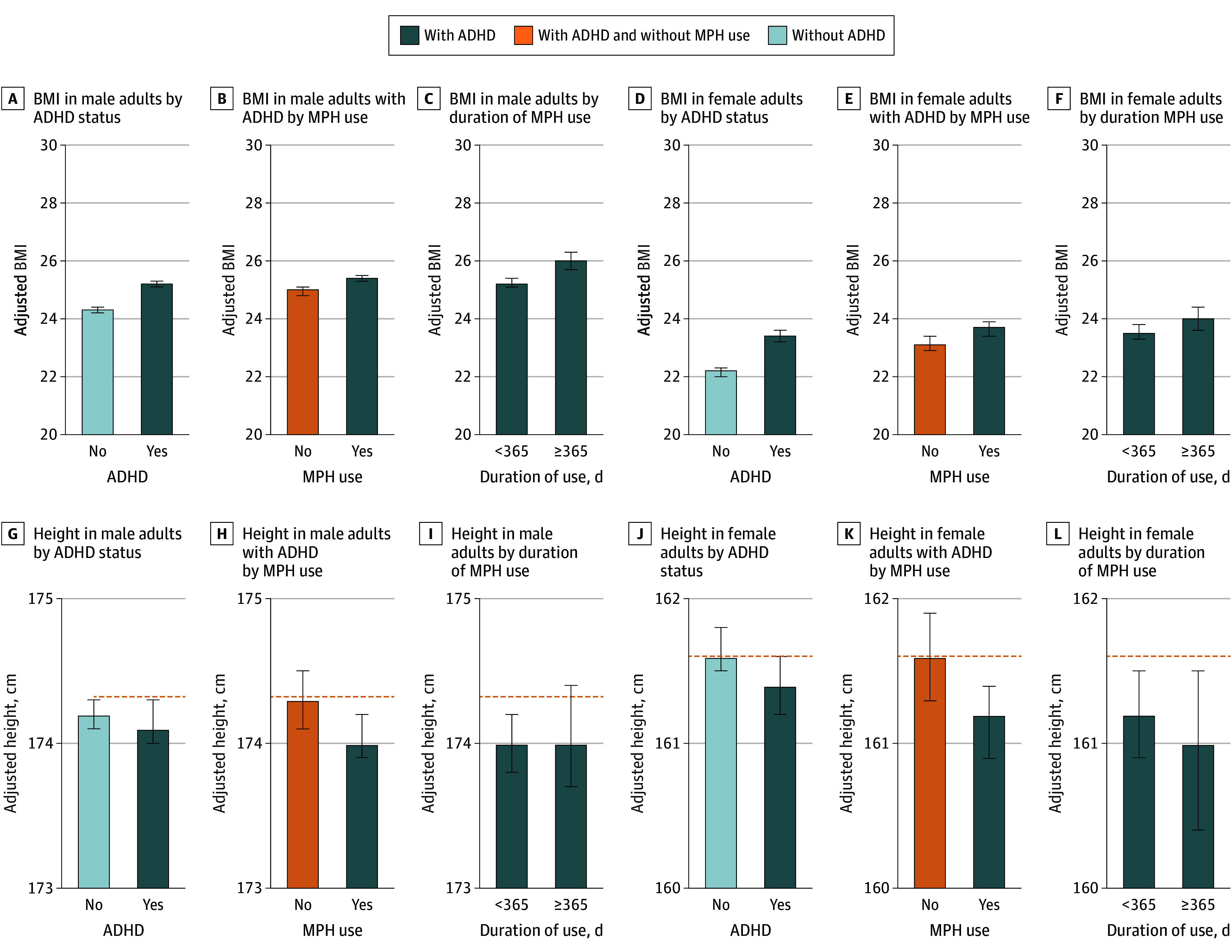
Body Mass Index (BMI) and Height at Adulthood by Prevalence of Attention-Deficit/Hyperactivity Disorder (ADHD) and Methylphenidate (MPH) Use Among Children Adjusted means of BMI (calculated as weight in kilograms divided by height in meters squared) and height (in centimeters) with 95% CIs (error bars) at adulthood (aged 20-25 years) were calculated by linear regression with the adjustments of age, sex, income level, Charlson Comorbidity Index, other drug prescriptions, and time to the health checkup among children (aged 6-11 years), according to the prevalence of ADHD, MPH use, and cumulative usage (in days). Orange dashed lines indicate adjusted height in individuals without ADHD.

The prevalence of overweight and obesity was 35.0% in the group without ADHD, 44.9% in the group with ADHD, and 46.5% in the group treated with MPH (Table). In model 2, compared with that in the group without ADHD, the AORs for overweight were 1.51 (95% CI, 1.44-1.59; *P* < .001) in the group with ADHD, 1.43 (95% CI, 1.34-1.52; *P* < .001) in the group without MPH, and 1.60 (95% CI, 1.51-1.71; *P* < .001) in group with MPH. The risk for overweight and obesity in those receiving MPH was higher than that in those not receiving MPH (1.13 [95% CI, 1.05-1.21]; *P* = .001). For severe obesity (BMI ≥30), the prevalence was significantly higher in the group with ADHD (14.9%) compared with that in the group without ADHD (9.3%) (*P* < .001). This proportion increased to 16.1% in those receiving MPH (AOR, 1.88 [95% CI, 1.71-2.06]; *P* < .001). These differences remained statistically significant after adjustment (*P* < .001 for trend in model 2). The AOR in model 2 for short stature was 1.08 (95% CI, 1.02-1.15; *P* = .01) for those receiving MPH. Sex-stratified analyses showed consistent findings in both males and females (eTable 6 in Supplement 1). Age-stratified analyses also supported that both BMI-elevation and height-reduction patterns persisted across different follow-up age groups (eTable 7 in Supplement 1). These associations were similar but weaker in adolescents who had nearly completed physical growth, were newly diagnosed with ADHD, and were receiving MPH (eTable 8 in Supplement 1).

**Table.** Association of ADHD Prevalence With or Without MPH Use With BMI and Height at Adulthood, Among Children^a^

Outcome at adulthood	Children					
	Without ADHD	With ADHD				
		Total	No MPH use	MPH use		
				All	Cumulative use <365 d	Cumulative use 365-1460 d
**BMI**						
Overweight and obesity, No. (%)^b^	4506 (35.0)	5780 (44.9)	2614 (43.2)	3166 (46.5)	2227 (44.9)	939 (50.5)
AOR (95% CI)						
Model 1	1 [Reference]	1.52 (1.44-1.59)	1.43 (1.34-1.52)	1.60 (1.51-1.70)	1.51 (1.41-1.61)	1.87 (1.69-2.06)
*P* value	NA	<.001	<.001	<.001	<.001	<.001
Model 2 (main)	1 [Reference]	1.51 (1.44-1.59)	1.43 (1.34-1.52)	1.60 (1.51-1.71)	1.51 (1.41-1.62)	1.89 (1.70-2.09)
*P* value	NA	<.001	<.001	<.001	<.001	<.001
AOR (95% CI)	NA	NA	1 [Reference]	1.13 (1.05-1.21)	NA	NA
*P* value	NA	NA	NA	.001	NA	NA
AOR (95% CI)	NA	NA	NA	NA	1 [Reference]	1.25 (1.12-1.39)
*P* value	NA	NA	NA	NA	NA	<.001
Model 3	1 [Reference]	1.49 (1.41-1.57)	1.42 (1.33-1.51)	1.58 (1.48-1.69)	1.50 (1.40-1.62)	1.87 (1.68-2.08)
*P* value	NA	<.001	<.001	<.001	<.001	<.001
Model 4	1 [Reference]	1.51 (1.43-1.59)	1.42 (1.34-1.52)	1.60 (1.50-1.70)	1.51 (1.41-1.61)	1.88 (1.70-2.08)
*P* value	NA	<.001	<.001	<.001	<.001	<.001
Severe obesity (BMI ≥30), No. (%)	1190 (9.3)	1916 (14.9)	821 (13.6)	1095 (16.1)	740 (14.9)	355 (19.1)
AOR (95% CI), model 2	1 [Reference]	1.73 (1.60-1.87)	1.57 (1.43-1.73)	1.88 (1.71-2.06)	1.74 (1.57-1.92)	2.29 (2.01-2.63)
*P* value	NA	<.001	<.001	<.001	<.001	<.001
AOR (95% CI)	NA	NA	1 [Reference]	1.20 (1.08-1.32)	NA	NA
*P* value	NA	NA	NA	<.001	NA	NA
AOR (95% CI)	NA	NA	NA	NA	1 [Reference]	1.32 (1.15-1.52)
*P* value	NA	NA	NA	NA	NA	<.001
**Height**						
Short stature, No. (%)^c^	6560 (51.0)	6673 (51.9)	3057 (50.5)	3616 (53.1)	2625 (53.0)	991 (53.3)
AOR (95% CI)						
Model 1	1 [Reference]	1.04 (0.99-1.09)	0.98 (0.92-1.04)	1.09 (1.02-1.15)	1.08 (1.01-1.16)	1.10 (0.99-1.21)
*P* value	NA	.16	.56	.006	.02	.06
Model 2 (main)	1 [Reference]	1.03 (0.98-1.08)	0.98 (0.92-1.04)	1.08 (1.02-1.15)	1.08 (1.01-1.15)	1.10 (0.99-1.21)
*P* value	NA	.28	.42	.01	.03	.08
AOR (95% CI)	NA	NA	NA	1.11 (1.04-1.19)	NA	NA
*P* value	NA	NA	NA	.003	NA	NA
AOR (95% CI)	NA	NA	NA	NA	1 [Reference]	1.02 (0.91-1.13)
*P* value	NA	NA	NA	NA	NA	.78
Model 3	1 [Reference]	1.03 (0.97-1.08)	0.98 (0.92-1.04)	1.09 (1.02-1.17)	1.09 (1.01-1.17)	1.11 (0.99-1.23)
*P* value	NA	.34	.06	.45	.02	.06
Model 4	1 [Reference]	1.02 (0.97-1.07)	0.97 (0.91-1.03)	1.07 (1.01-1.14)	1.07 (1.00-1.15)	1.08 (0.98-1.19)
*P* value	NA	.44	.31	.02	.05	.13

Abbreviations: ADHD, attention-deficit/hyperactivity disorder; AOR, adjusted odds ratio; BMI, body mass index (calculated as weight in kilograms divided by height in meters squared); MPH, methylphenidate; NA, not applicable.

^a^ Adulthood refers to ages 20 to 25 years; children, ages 6 to 11 years. AORs, 95% CIs, and *P* values were calculated by logistic regression with the following adjustments and covariates: model 1: age, sex, and income level; model 2: model 1 plus Charlson Comorbidity Index, other drug prescriptions, and time to the health checkup; model 3: model 2 plus antidepressive drug prescriptions; and model 4: model 2 plus drinking habits, smoking status, and physical activity.

^b^ Calculated as a BMI of 25 or more for males and of 23 or more for females.

^c^ Calculated as less than 174.4 cm for males (aged 20-29 years) and as less than 161.8 cm for females (aged 20-29 years).

## Discussion

In this retrospective cohort study with approximately 12 years of follow-up among children and adolescents, we found that individuals diagnosed with ADHD, particularly those treated with MPH, exhibited higher BMI and slightly shorter height at adulthood compared with matched controls without ADHD. These patterns were more apparent among those with prolonged MPH therapy exposure (≥365 days), in whom the highest risks for obesity and height below the sex-specific standard means were observed. Notably, the risk was attenuated in adolescents who reached adulthood earlier than prepubertal children, suggesting that the potential association of MPH with obesity and shorter height may be more relevant to long-term use during childhood.

As few studies, to our knowledge, have shown that MPH use with ADHD is associated with BMI and height, the association of different ADHD-related or MPH-related lifestyles, especially with height, remains unclear. Nonetheless, our findings support the hypothesis that ADHD and long-term MPH use may be risk factors for obesity and short stature. The potential mechanisms may partially overlap. Both outcomes may be influenced by behavioral dysregulation and altered neurophysiological conditions, such as fluctuating neurotransmitter activity. Height in adulthood is primarily influenced by alterations in growth velocity, which may be affected by the duration of MPH exposure, whereas differences in BMI mainly reflect metabolic and energy balance factors. While BMI demonstrated a consistent dose-response association with both adjusted mean and total MPH dose, the MPH dose-response risk was lower for short stature with height. Because growth may be sensitive to lifestyle and behavioral changes associated with prolonged MPH use, long-term exposure might lead to sustained unhealthy habits, which could partly explain the dose-response risk being lower for height deficit.

Pediatric patients with ADHD commonly experience chronic stress, disrupted sleep, and circadian rhythm disturbances, which may dysregulate hormonal systems and may adversely affect energy balance and growth.^22,23,24,25^ Moreover, MPH acts by inhibiting dopamine and norepinephrine reuptake in the central nervous system, increasing their synaptic concentrations.^26^ As dopamine plays a key role, not only in behavior and cognition but also in growth regulation via the hypothalamic-pituitary-axis,^27^ fluctuating dopamine levels and unstable growth control in children with ADHD and MPH use might negatively influence growth.^28^ For instance, animal models and neuroendocrine studies have demonstrated that dopaminergic stimulation can suppress growth hormone secretion,^29,30^ which may partially explain the observed shorter height in those treated with MPH. Similarly, several short-term and medium-term studies have reported that MPH use could influence early growth; a notable prospective study from the Multimodal Treatment Study of Children With ADHD trial found that initial height suppression with stimulants was not fully recovered even after several years.^31,32^ Although MPH use was associated with lower risk of hormonal regulation and shorter height, previous research reported persistent height deficits in children treated with MPH.^33^ Additionally, MPH is well known to reduce appetite, particularly in the initial stages of treatment. This appetite suppression may lead to slow height growth velocity with reduced daily caloric intake.^17^

Our study found associations of ADHD and MPH use with overweight and obesity (higher BMI) and short stature using a large-scale, representative cohort. Despite these findings, the clinical relevance should be interpreted with caution. In our cohort, the mean difference in height was less than 1 cm (eg, maximum −0.6 cm in females) below commonly accepted thresholds for clinical significance (typically an SD of 4-5 cm or 2 times the SD: approximately 10 cm).^34,35,36^ In addition, the PES value was less than .001, indicating a small effect size with weak Pearson correlation coefficients, particularly for height; their clinical correlation appears modest. Nevertheless, the observed duration-response association suggests that MPH may contribute, even slightly, to long-term changes in body composition and growth. These findings do not suggest that MPH use should be discontinued, especially given its well-established efficacy in managing ADHD symptoms.^37^ Rather, we suggest the importance of balanced clinical decision-making that considers both benefits and potential long-term risks. Clinicians should be aware of its potential association with growth trajectories. Routine monitoring of weight and height, along with counseling on nutrition and physical activity, may help mitigate adverse outcomes. To promote optimal growth, management strategies such as improved sleep quality with sufficient sleep duration, balanced nutritional intake, and regular physical activity should be considered, rather than modifying treatment strategies. For children at higher risk, such as those with low birth weight, delayed puberty, or poor nutritional status, moreover, alternative dosing strategies or nonstimulant treatments might be considered. From a public health perspective, our results suggest the need for long-term surveillance of physical health outcomes in populations with ADHD, particularly those receiving long-term pharmacologic therapy.

### Limitations

Some limitations of this study should be noted. First, residual confounding, including parents’ BMI and height, pubertal or menstrual status, genetic variance, nutritional intake and status, sleep time and quality, heart rate, and serum concentration of growth hormone, and time-varying factors and confounding due to long-term follow-up cannot be entirely considered despite feasible collection and covariate adjustment. Notably, as our study was conducted on a population in Asia, the possibility of differences according to race was suggested. Second, as both patients with and without ADHD were required to have undergone at least 1 health examination (from 2018 to 2022), the analytic population was inherently restricted to health-examination participants. Although this condition was necessary to obtain measures of BMI and height at adulthood, inclusion criteria may have introduced selection bias; individuals who completed health examinations might differ systematically in health behaviors or socioeconomic characteristics from those who did not. Third, as intermediate data on BMI, height, lifestyle behaviors, and nutritional status in children and adolescents were unavailable, changes in BMI and height between childhood and early adulthood and the mediation association with lifestyles could not be evaluated. Prescribed dosage is usually related to weight and height at childhood and adolescence, which were not considered in the dose analysis, representing a limitation. It was also not possible to evaluate how obesity-related lifestyles may have contributed to shorter height. Further studies with detailed assessments of lifestyles, including physical activity and sleep patterns, are needed to clarify these mechanisms. Next, this retrospective cohort study relied on claims-based operational definitions for ADHD diagnosis and MPH use. The possibility of misclassification or diagnostic ambiguity cannot be fully excluded because the codes may not perfectly reflect clinical assessments. Our study could not fully account for the complex and heterogeneous patterns of MPH use, including variations in schedules, treatment interruptions, changes over the 4-year follow-up period, or discontinuity. As prescription claims capture only dispensed medications and not actual adherence or intermittent-use patterns, the cumulative exposure may not fully reflect the diversity of medication behaviors in the general population. Additionally, while we observed associations, causality cannot be definitively established given the observational nature of the study, indicating the need for additional long-term clinical studies to support our findings.

## Conclusions

The findings of this cohort study suggest that prepubertal children and adolescents with ADHD, especially those treated with MPH, may be at risk for higher BMI and shorter height in adulthood. While the benefits of MPH in managing ADHD symptoms are well established, the findings suggest a need for balanced clinical decision-making that considers potential long-term physical consequences such as obesity and short stature. Future research should focus on identifying modifiable risk factors, optimizing treatment strategies, and developing comprehensive guidelines for monitoring growth and metabolic health in populations with ADHD.

## Notes

Supplement 1. **eFigure 1.** Study Design **eFigure 2.** Flow Diagram for the Study Population **eFigure 3.** Distribution of Methylphenidate (MPH) Use (Days of Use or Dose) Among Children With Attention-Deficit/Hyperactivity Disorder (ADHD) and MPH **eTable 1.** Baseline Characteristics of Children **eTable 2.** Baseline Characteristics of Adolescents **eTable 3.** Average BMI/Height at Adulthood, According to the Prevalence of ADHD and MPH Use Among Children **eTable 4.** Average Height With Stratification of BMI at Adulthood, According to the Prevalence of ADHD and MPH Use Among Children **eTable 5.** Average BMI/Height at Adulthood, According to the Prevalence of ADHD and MPH Use Among Adolescents **eFigure 4.** Scatterplot Between the Cumulative Days to MPH Use and Body Mass Index (BMI)/Height at Adulthood Among MPH Users (Children; Age = 6-11 Years) With ADHD **eFigure 5.** BMI and Height at Adulthoods, According to Total Dose of MPH Use (Cumulative; <7000 mg or ≥7000 mg), Among MPH Users With ADHD **eTable 6.** Association of the Prevalence of ADHD and MPH Use With BMI/Height at Adulthood by Sex of Children **eTable 7.** Association of the Prevalence of ADHD and MPH Use With BMI/Height at Adulthood, Among Children **eTable 8.** Association of the Prevalence of ADHD and MPH Use With BMI/Height at Adulthood, Among Adolescents

Supplement 2.
Data Sharing Statement
