# Supplementary material for: ADHD and Methylphenidate Use in Prepubertal Children and BMI and Height at Adulthood
Source: JAMA Netw Open. 2026 Jan 5;9(1):e2552019. doi: 10.1001/jamanetworkopen.2025.52019 (PMC12771255; doi:10.1001/jamanetworkopen.2025.52019)
Supplement: Supplement 1. — eFigure 1. Study Design eFigure 2. Flow Diagram for the Study Population eFigure 3. Distribution of Methylphenidate (MPH) Use (Days of Use or Dose) Among Children With Attention-Deficit/Hyperactivity Disorder (ADHD) and MPH eTable 1. Baseline Characteristics of Children eTable 2. Baseline Characteristics of Adolescents eTable 3. Average BMI/Height at Adulthood, According to the Prevalence of ADHD and MPH Use Among Children eTable 4. Average Height With Stratification of BMI at Adulthood, According to the Prevalence of ADHD and MPH Use Among Children eTable 5. Average BMI/Height at Adulthood, According to the Prevalence of ADHD and MPH Use Among Adolescents eFigure 4. Scatterplot Between the Cumulative Days to MPH Use and Body Mass Index (BMI)/Height at Adulthood Among MPH Users (Children; Age = 6-11 Years) With ADHD eFigure 5. BMI and Height at Adulthoods, According to Total Dose of MPH Use (Cumulative; <7000 mg or ≥7000 mg), Among MPH Users With ADHD eTable 6. Association of the Prevalence of ADHD and MPH Use With BMI/Height at Adulthood by Sex of Children eTable 7. Association of the Prevalence of ADHD and MPH Use With BMI/Height at Adulthood, Among Children eTable 8. Association of the Prevalence of ADHD and MPH Use With BMI/Height at Adulthood, Among Adolescents [file jamanetwopen-e2552019-s001.pdf]

Supplemental Online Content

Song J, Park SJ, Yu J, Chung J, Jeong S, Park SM. ADHD and methylphenidate use in prepubertal children and BMI and height at adulthood. *JAMA Netw Open*. 2026;8(1):e2552019. doi:10.1001/jamanetworkopen.2025.52019

eFigure 1. Study Design
eFigure 2. Flow Diagram for the Study Population
eFigure 3. Distribution of Methylphenidate (MPH) Use (Days of Use or Dose) Among Children With Attention-Deficit/Hyperactivity Disorder (ADHD) and MPH
eTable 1. Baseline Characteristics of Children
eTable 2. Baseline Characteristics of Adolescents
eTable 3. Average BMI/Height at Adulthood, According to the Prevalence of ADHD and MPH Use Among Children
eTable 4. Average Height With Stratification of BMI at Adulthood, According to the Prevalence of ADHD and MPH Use Among Children
eTable 5. Average BMI/Height at Adulthood, According to the Prevalence of ADHD and MPH Use Among Adolescents
eFigure 4. Scatterplot Between the Cumulative Days to MPH Use and Body Mass Index (BMI)/Height at Adulthood Among MPH Users (Children; Age = 6-11 Years) With ADHD
eFigure 5. BMI and Height at Adulthoods, According to Total Dose of MPH Use (Cumulative; <7000 mg or ≥7000 mg), Among MPH Users With ADHD
eTable 6. Association of the Prevalence of ADHD and MPH Use With BMI/Height at Adulthood by Sex of Children
eTable 7. Association of the Prevalence of ADHD and MPH Use With BMI/Height at Adulthood, Among Children
eTable 8. Association of the Prevalence of ADHD and MPH Use With BMI/Height at Adulthood, Among Adolescents

This supplemental material has been provided by the authors to give readers additional information about their work.

© 2026 Song J et al. *JAMA Network Open*.

eFigure 1. Study Design

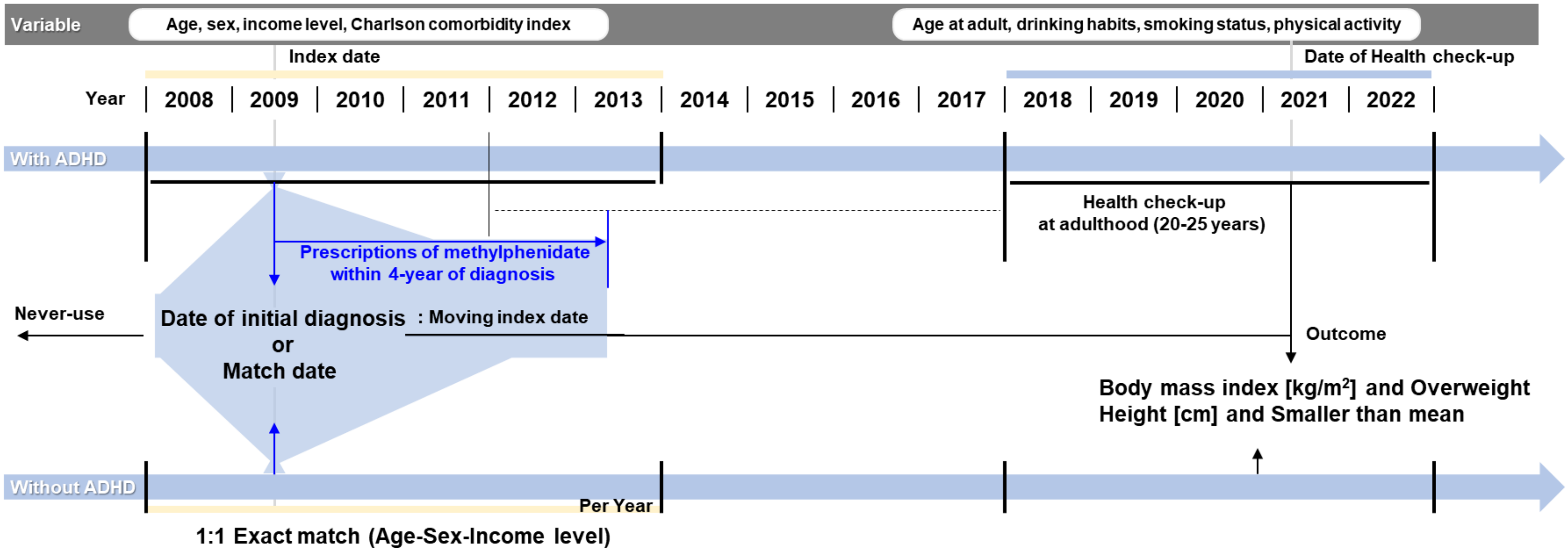

© 2026 Song J et al. *JAMA Network Open*.

eFigure 2. Flow Diagram for the Study Population

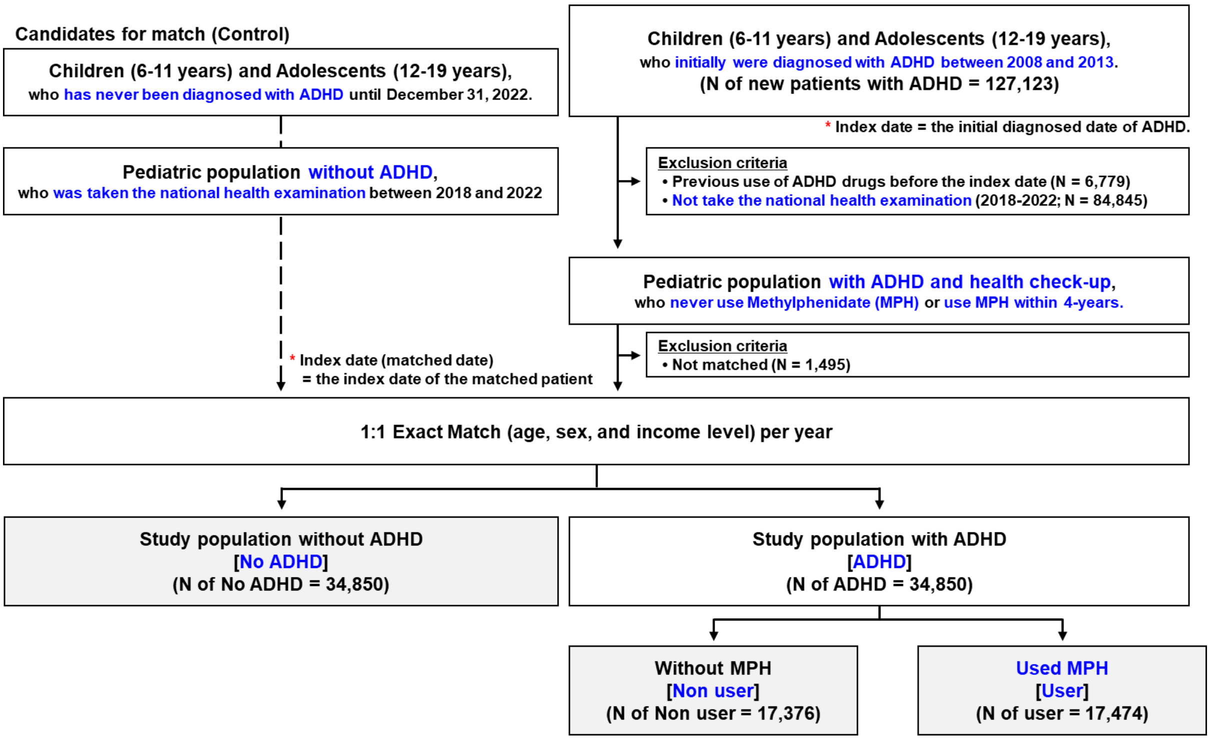

© 2026 Song J et al. *JAMA Network Open*.

eFigure 3. Distribution of Methylphenidate (MPH) Use (Days of Use or Dose) Among Children With Attention-Deficit/Hyperactivity Disorder (ADHD) and MPH

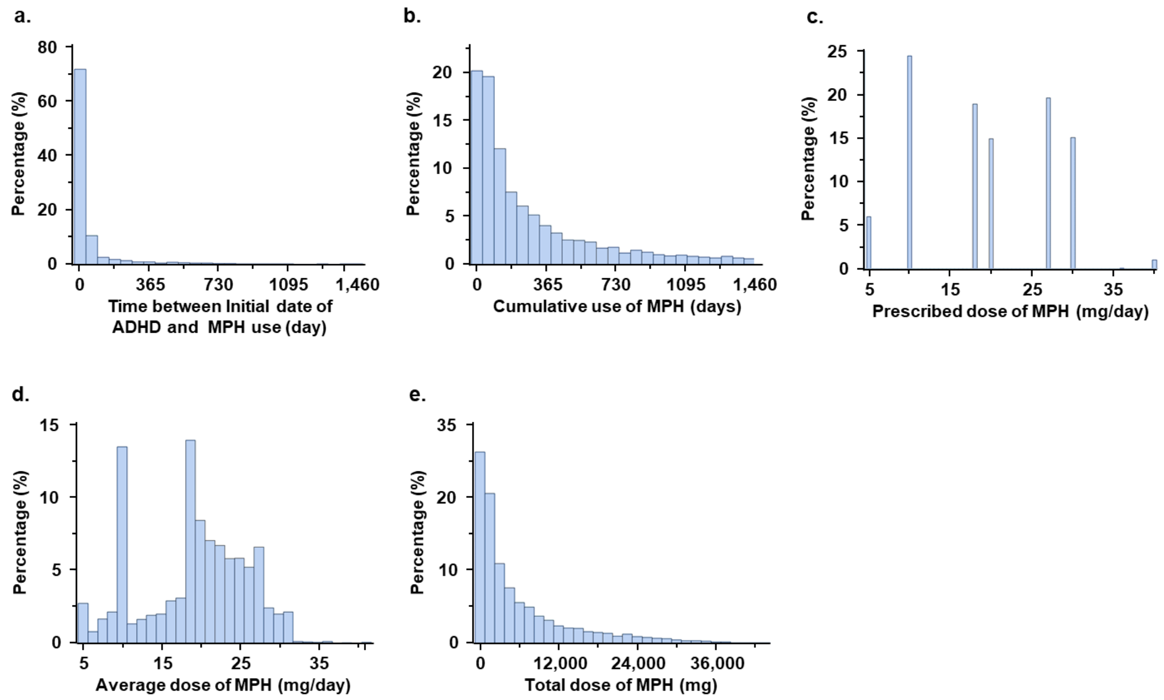

A. elapsed time from initial ADHD diagnosis to first prescription of MPH (median=10 days), **B.** total days of medication use within the 4-year period (median=140 days), **C.** daily dose in a prescription, **D.** average dose of MPH (median=19.6 mg/day), and **E.** total exposure dose of MPH within 4- year (median=2,565)

© 2026 Song J et al. *JAMA Network Open.*

eTable 1. Baseline Characteristics of Children

	Study population: children (6–12 years) at the year of ADHD			
	Without ADHD	With ADHD		
	No ADHD (Matched)	Whether to use MPH or not		
		ADHD (All)	Non user	User of MPH
**Study population, N**	12,866	12,866	6,050	6,816
**Duration of MPH use [days][a], mean (SD)**	0		0	285.2 (339.2)
**Duration of MPH use[a], N (%)**				
Use within a year (< 365 days)				4,956 (72.7)
Use within 1–4 years (≥ 365 days)				1,860 (27.3)
**Average dose of MPH [mg/day of use][a], N (%)**				
< 20				3,588 (52.6)
20 ≤, ≤ 40				3,228 (47.3)
**Total exposure dose [mg of total use][a], N (%)**				
< 7,000				4,911 (72.0)
≥ 7,000				1,905 (28.0)
**Another [Used][a], N (%)**				
Atomoxetine	0	1,197 (9.3)	307 (5.1)	890 (13.1)
Clonidine	0	20 (0.2)	4 (0.1)	16 (0.2)
**Anti-depressive drugs [Used][a], N (%)**	54 (0.4)	2,673 (20.8)	429 (7.1)	2,244 (32.9)
**Selective Serotonin Reuptake Inhibitors**	15 (0.1)	891 (6.9)	134 (2.2)	757 (11.1)
**Age [year] at start[b], mean (SD)**	9.3 (1.4)	9.3 (1.4)	9.4 (1.3)	9.2 (1.4)
**Time to the health check-up [year], mean (SD)**	11.8 (1.6)	11.7 (1.5)	11.6 (1.6)	11.8 (1.5)
**Age [year] at end[b], mean (SD)**	21.9 (1.5)	21.8 (1.5)	21.8 (1.4)	21.8 (1.5)
**Sex, N (%)**				
Male	9,329 (72.5)	9,329 (72.5)	4,319 (71.4)	5,010 (73.5)
Female	3,537 (27.5)	3,537 (27.5)	1,731 (28.6)	1,806 (26.5)
**Household income, N (%)**				
Q1 (High)	5,018 (39.0)	5,018 (39.0)	2,497 (41.3)	2,521 (37.0)
Q2	3,747 (29.1)	3,748 (29.1)	1,772 (29.3)	1,976 (29.0)
Q3	2,178 (16.9)	2,176 (16.9)	954 (15.8)	1,222 (17.9)
Q4 (Low)	1,923 (15.0)	1,924 (15.0)	827 (13.7)	1,097 (16.1)
**Charlson comorbidity index, N (%)**				
0	9,772 (76.0)	8,881 (69.0)	4,174 (69.0)	4,707 (69.1)
1-2	3,063 (23.8)	3,919 (30.5)	1,847 (30.5)	2,072 (30.4)
≥ 3	31 (0.2)	66 (0.5)	29 (0.5)	37 (0.5)

[a]Using the history of prescription within four years from the initial diagnosis of ADHD.
[b]Age at start of follow-up (the date of diagnosis or match) or end of follow-up (the date of health check-up).

© 2026 Song J et al. *JAMA Network Open*.

Abbreviation: attention deficit hyperactivity disorder (ADHD), methylphenidate (MPH), number (N), standard deviation (SD).

© 2026 Song J et al. *JAMA Network Open*.

eTable 2. Baseline Characteristics of Adolescents

	Study population: adolescents (12–19 years) at the year of ADHD			
	Without ADHD	With ADHD		
	No ADHD (Matched)	Whether to use MPH or not		
		ADHD (All)	Non user	User of MPH
**Study population, N**	21,984	21,984	11,326	10,658
**Duration of MPH use [days][a], mean (SD)**				187.8 (264.5)
**Duration of MPH use[a], N (%)**				
Use within a year (< 365 days)				9,018 (84.6)
Use within 1–4 years (≥ 365 days)				1,640 (16.4)
**Average dose of MPH [mg/day of use][a], N (%)**				
< 20				5,253 (49.2)
20 ≤, ≤ 40				5,405 (50.7)
**Total exposure dose [mg of total use][a], N (%)**				
< 7,000				8,875 (83.3)
≥ 7,000				1,783 (16.7)
**Another [Used][a], N (%)**				
Atomoxetine	0	1,324 (6.0)	503 (4.4)	821 (7.7)
Clonidine	0	8 (0.0)	2 (0.0)	6 (0.1)
**Anti-depressive drugs [Used][a], N (%)**	295 (1.3)	6,888 (31.3)	1,896 (16.7)	4,992 (46.8)
**Selective Serotonin Reuptake Inhibitors**	108 (0.5)	2,787 (12.7)	814 (7.2)	1,973 (18.5)
**Age [year] at start[b], mean (SD)**	14.5 (1.8)	14.5 (1.8)	14.5 (1.8)	14.4 (1.8)
**Time to the health check-up [year], mean (SD)**	10.1 (2.0)	10.1 (2.0)	10.0 (2.0)	10.2 (2.0)
**Age [year] at end[b], mean (SD)**	24.4 (2.3)	24.4 (2.4)	24.3 (2.4)	24.5 (2.4)
**Sex, N (%)**				
Male	14,633 (66.6)	14,633 (66.6)	7,586 (67.0)	7,047 (66.1)
Female	7,351 (33.4)	7,351 (33.4)	3,740 (33.0)	3,611 (33.9)
**Household income, N (%)**				
Q1 (High)	11,341 (51.6)	11,341 (51.6)	5,970 (52.7)	5,371 (50.4)
Q2	4,705 (21.4)	4,705 (21.4)	2,424 (21.4)	2,281 (21.4)
Q3	2,960 (13.5)	2,960 (13.5)	1,489 (13.2)	1,471 (13.8)
Q4 (Low)	2,978 (13.6)	2,978 (13.6)	1,443 (12.7)	1,535 (14.4)
**Charlson comorbidity index, N (%)**				
0	20,659 (94.0)	16,461 (74.9)	8,466 (74.8)	7,995 (75.0)
1-2	1,300 (5.9)	5,340 (24.3)	2,766 (24.4)	2,574 (24.2)
≥ 3	25 (0.1)	183 (0.8)	94 (0.8)	89 (0.8)

[a]Using the history of prescription within four years from the initial diagnosis of ADHD.

[b]Age at start of follow-up (the date of diagnosis or match) or end of follow-up (the date of health check-up).

© 2026 Song J et al. *JAMA Network Open*.

Abbreviation: attention deficit hyperactivity disorder (ADHD), methylphenidate (MPH), number (N), standard deviation (SD).

© 2026 Song J et al. *JAMA Network Open*.

eTable 3. Average BMI/Height at Adulthood, According to the Prevalence of ADHD and MPH Use Among Children

	Children (6-11 years)					
	No ADHD	With ADHD				
		ADHD (All)	No user	User of MPH, cumulative usage [days of use]		
				User (All)	<365	365-1,460
**BMI at adulthood (20–25 years)**						
Crude mean (SD) [kg/m^2]						
All	23.6 (4.6)	24.6 (5.2)	24.3 (5.0)	24.9 (5.3)	24.7 (5.3)	25.4 (5.4)
Male	24.2 (4.7)	25.1 (5.2)	24.9 (5.0)	25.3 (5.3)	25.1 (5.3)	25.8 (5.4)
Female	22.0 (3.9)	23.3 (5.0)	23.1 (4.8)	23.6 (5.2)	23.5 (5.1)	23.9 (5.4)
Adjusted mean (95% CIs) [kg/m^2]						
All	23.3 (23.2, 23.4)	24.3 (24.2, 24.4)#	24.0 (23.9, 24.2)#	24.5 (24.4, 24.6)#	24.3 (24.2, 24.5)#	25.0 (24.8, 25.2)#
p-value	Reference	<.001	<.001	<.001	<.001	<.001
PES	.010 (ADHD)		.011 (Use of MPH)		.011 (Cumulative use)	
Male	24.3 (24.2, 24.4)	25.2 (25.1, 25.3)#	25.0 (24.8, 25.1)#	25.4 (25.3, 25.6)#	25.2 (25.1, 25.4)#	26.0 (25.7, 26.3)#
Female	22.2 (22.0, 22.3)	23.4 (23.2, 23.6)#	23.1 (22.9, 23.4)#	23.7 (23.4, 23.9)#	23.5 (23.3, 23.8)#	24.0 (23.6, 24.4)#
**Height at adulthood (20–25 years)**						
Crude mean (SD) [cm]						
All	170.8 (8.1)	170.6 (8.4)	170.7 (8.4)	170.6 (8.4)	170.5 (8.4)	170.9 (8.4)
Male	174.3 (6.0)	174.1 (6.3)	174.3 (6.3)	174.0 (6.3)	174.0 (6.3)	174.0 (6.4)
Female	161.7 (5.4)	161.4 (5.7)	161.6 (5.5)	161.2 (5.8)	161.3 (5.8)	161.0 (5.7)
Adjusted mean (95% CIs) [cm]						
All	167.9 (167.8, 168.0)	167.8 (167.7, 167.9)	167.9 (167.8, 168.1)	167.6 (167.5, 167.8)**	167.6 (167.5, 167.8)*	167.6 (167.3, 167.9)*
p-value	Reference	.099	.808	.004	.011	.045
PES	.0001 (ADHD)		.0004 (Use of MPH)		.0004 (Cumulative use)	
Male	174.2 (174.1, 174.3)	174.1 (174.0, 174.3)*	174.3 (174.1, 174.5)	174.0 (173.9, 174.2)**	174.0 (173.8, 174.2)*	174.0 (173.7, 174.4)*
Female	161.6 (161.5, 161.8)	161.4 (161.2, 161.6)*	161.6 (161.3, 161.9)	161.2 (160.9, 161.4)**	161.2 (160.9, 161.5)**	161.0 (160.4, 161.5)*

Adjusted mean, 95% CIs, and *p*-value were calculated by Linear regression with the following adjustments and covariates: age, sex, income level, Charlson comorbidity index, other drug prescriptions, and time to the health check-up.

Abbreviation: attention deficit hyperactivity disorder (ADHD), methylphenidate (MPH), body mass index (BMI), number (N), confidence intervals (CIs), proportion of explained variance (PES).

**p*-value<.05, ***p*-value<.01, and #*p*-value<.001, compared to No ADHD.

© 2026 Song J et al. *JAMA Network Open*.

eTable 4. Average Height With Stratification of BMI at Adulthood, According to the Prevalence of ADHD and MPH Use Among Children

	Children (6-11 years)					
	No ADHD	With ADHD				
		ADHD (All)	No user	User of MPH, cumulative usage [days of use]		
				User (All)	<365	365-1,460
**Height at adulthood (20–25 years)**						
1. Overweight and obesity (20–25 years)						
Crude mean (SD) [cm]						
Male	174.7 (6.0)	174.5 (6.5)	174.7 (6.6)	174.3 (6.4)	174.3 (6.3)	174.3 (6.6)
Female	161.9 (5.6)	161.2 (6.0)	161.5 (5.9)	161.0 (6.1)	161.0 (6.2)	161.1 (5.9)
Adjusted mean (95% CIs) [cm]						
Male	174.7 (174.5, 174.9)	174.5 (174.3, 174.7)	174.7 (174.4, 174.9)	174.3 (174.1, 174.6)*	174.3 (174.0, 174.6)	174.3 (173.8, 174.7)
p-value	Reference	.176	.898	.041	.139	.179
Female	161.9 (161.5, 162.2)	161.2 (160.9, 161.5)**	161.4 (161.0, 161.9)	161.0 (160.6, 161.5)**	161.0 (160.5, 161.5)	161.1 (160.3, 161.9)
p-value	Reference	.008	.134	.003	.183	.467
**2. Severe obesity (20–25 years)**						
Crude mean (SD) [cm]						
Male	175.3 (5.9)	175.0 (6.8)	175.2 (6.7)	174.8 (6.8)	175.0 (6.9)	174.4 (6.5)
Female	163.3 (5.0)	161.4 (6.5)	162.0 (6.2)	160.9 (6.8)	160.8 (6.6)	161.4 (7.1)
Adjusted mean (95% CIs) [cm]						
Male	175.3 (174.9, 175.7)	174.9 (174.6, 175.3)	175.2 (174.7, 175.7)	174.8 (174.3, 175.2)	175.0 (174.5, 175.5)	174.3 (173.6, 175.0)*
p-value		.144	.612	.063	.317	.018
Female	163.2 (162.2, 164.1)	161.5 (160.8, 162.1)**	161.2 (161.1, 163.0)*	161.0 (160.2, 161.9)#	160.9 (159.9, 161.8)#	161.6 (159.9, 163.2)
p-value		.003	.010	<.001	<.001	.087

Stratification of BMI at adulthood: overweight and obesity (≥25.0 kg/m^2 for male and ≥23.0 kg/m^2 for female) and severe obesity (≥30.0 kg/m^2 for both).

Adjusted mean, 95% CIs, and *p*-value were calculated by Linear regression with the following adjustments and covariates: age, sex, income level, Charlson comorbidity index, other drug prescriptions, and time to the health check-up.

Abbreviation: attention deficit hyperactivity disorder (ADHD), methylphenidate (MPH), body mass index (BMI), number (N), confidence intervals (CIs).

**p*-value<.05, ***p*-value<.01, and #*p*-value<.001, compared to No ADHD.

© 2026 Song J et al. *JAMA Network Open.*

eTable 5. Average BMI/Height at Adulthood, According to the Prevalence of ADHD and MPH Use Among Adolescents

	Adolescents (12-19 years) at the year of ADHD					
	No ADHD	With ADHD				
		ADHD (All)	No user	User of MPH, cumulative usage [days of use]		
				User (All)	<365	365-1,460
**BMI at adulthood (20–25 years)**						
Crude mean (SD) [kg/m^2]						
All	24.0 (4.3)	24.5 (4.9)	24.4 (4.9)	24.7 (4.9)	24.6 (4.9)	24.8 (5.0)
Male	24.8 (4.1)	25.4 (4.8)	25.2 (4.8)	25.5 (4.8)	25.4 (4.8)	25.8 (5.0)
Female	22.2 (4.2)	22.9 (4.8)	22.8 (4.7)	23.0 (4.8)	23.1 (4.9)	22.9 (4.5)
Adjusted mean (95% CIs) [kg/m^2]						
All	23.7 (23.6, 23.7)	24.2 (24.2, 24.3)#	24.1 (24, 24.2)#	24.4 (24.3, 24.4)#	24.3 (24.2, 24.4)#	24.6 (24.4, 24.8)#
p-value	Reference	<.001	<.001	<.001	<.001	<.001
Male	24.9 (24.8, 25.0)	25.4 (25.3, 25.5)#	25.3 (25.2, 25.4)#	25.5 (25.4, 25.7)#	25.5 (25.4, 25.6)#	25.9 (25.7, 26.2)#
Female	22.4 (22.3, 22.5)	23.1 (23.0, 23.2)#	23.0 (22.8, 23.1)#	23.2 (23.1, 23.4)#	23.2 (23.1, 23.4)#	23.2 (22.8, 23.5)#
**Height at adulthood (20–25 years)**						
Crude mean (SD) [cm]						
All	170.4 (8.3)	170.3 (8.5)	170.4 (8.5)	170.2 (8.5)	170.2 (8.5)	170.1 (8.6)
Male	174.7 (5.9)	174.6 (6.1)	174.6 (6.2)	174.6 (6.0)	174.6 (5.9)	174.4 (6.4)
Female	161.8 (5.4)	161.7 (5.5)	161.8 (5.5)	161.6 (5.5)	161.6 (5.4)	161.6 (5.6)
Adjusted mean (95% CIs) [cm]						
All	168.1 (168.1, 168.2)	168.0 (168.0, 168.1)	168.1 (168.0, 168.2)	168.0 (167.9, 168.1)*	168.0 (167.9, 168.2)	167.9 (167.6, 168.2)
p-value	Reference	.089	.373	.049	.108	.100
Male	174.6 (174.5, 174.7)	174.5 (174.4, 174.6)	174.5 (174.4, 174.7)	174.5 (174.4, 174.7)	174.5 (174.4, 174.7)	174.4 (174.0, 174.7)
Female	161.7 (161.5, 161.8)	161.5 (161.4, 161.7)*	161.6 (161.4, 161.8)	161.4 (161.2, 161.6)*	161.4 (161.2, 161.6)	161.4 (160.9, 161.8)

Adjusted mean, beta-coefficients, 95% CIs, and *p*-value were calculated by Linear regression with the following adjustments and covariates: age, sex, income level, Charlson comorbidity index, other drug prescriptions, and time to the health check-up.
Abbreviation: attention deficit hyperactivity disorder (ADHD), methylphenidate (MPH), body mass index (BMI), number (N), confidence intervals (CIs).
**p*-value<.05, ***p*-value<.01, and #*p*-value<.001, compared to No ADHD.

© 2026 Song J et al. *JAMA Network Open*.

eFigure 4. Scatterplot Between the Cumulative Days to MPH Use and Body Mass Index (BMI)/Height at Adulthood Among MPH Users (Children; Age = 6-11 Years) With ADHD

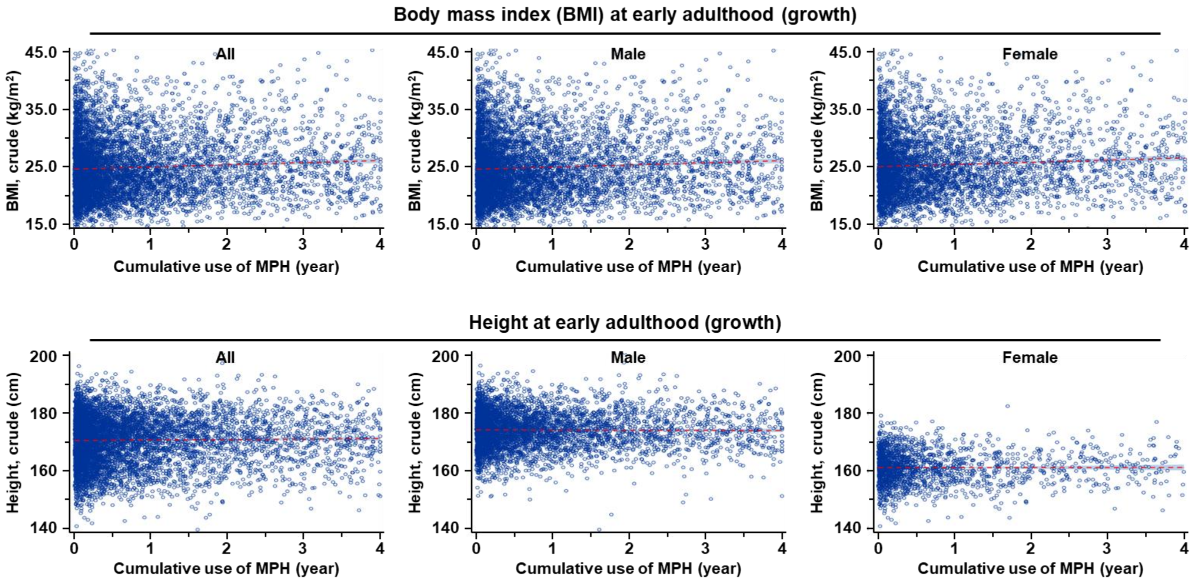

Association of the cumulative days of MPH use per the observed duration (4 years from the initial diagnosed date of ADHD) with BMI (upper; Pearson correlation coefficient=0.065) and height (lower; -0.018) in users of MPH. The red dashed line indicates the trend of optimized linear regression with 95% CIs

© 2026 Song J et al. *JAMA Network Open*.

eFigure 5. BMI and Height at Adulthoods, According to Total Dose of MPH Use (Cumulative; <7000 mg or ≥7000 mg), Among MPH Users With ADHD

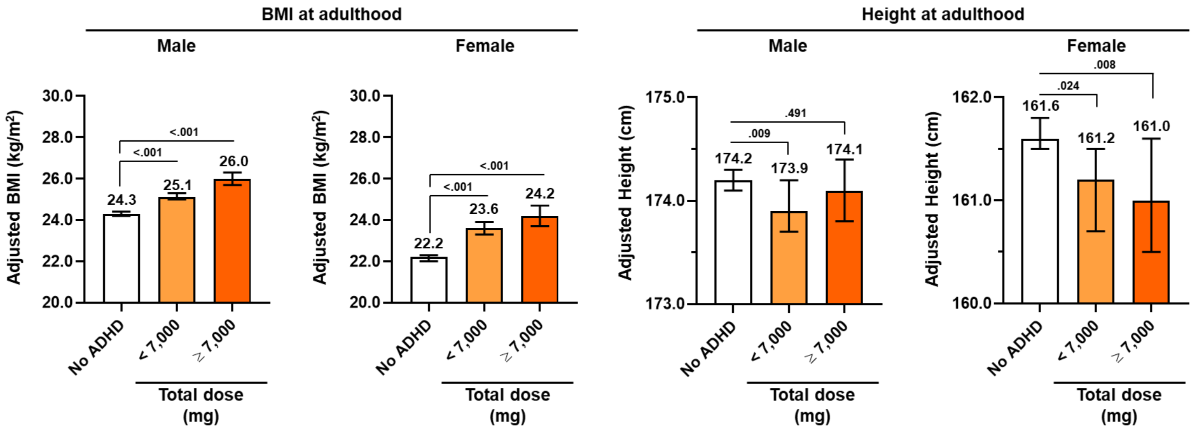

Adjusted mean, 95% CIs, and *p*-value were calculated by Linear regression with the following adjustments and covariates: age, sex, income level, Charlson comorbidity index, other drug prescriptions, and time to the health check-up

© 2026 Song J et al. *JAMA Network Open*.

eTable 6. Association of the Prevalence of ADHD and MPH Use With BMI/Height at Adulthood by Sex of Children

	Children (6-11 years) at the year of ADHD					
		With ADHD				
	No ADHD	ADHD (All)	No user	User of MPH, cumulative usage [days of use]		
				User (All)	<365	365-1,460
**BMI at adulthood (20–25 years)**						
**1. Male**						
Overweight and obesity, N (%)	3,367 (36.1)	4,228 (45.3)	1,887 (43.7)	2,341 (46.7)	1,619 (45.1)	722 (51.0)
aORs (95% CIs)	1.00 (Reference)	1.47 (1.39, 1.57)#	1.39 (1.29, 1.50)#	1.55 (1.45, 1.67)#	1.45 (1.34, 1.58)#	1.86 (1.66, 2.09)#
			1.00 (Reference)	1.12 (1.03, 1.22)**		
					1.00 (Reference)	1.28 (1.13, 1.45)#
**2. Female**						
Overweight and obesity, N (%)	1139 (32.2)	1,552 (43.9)	727 (42.0)	825 (45.7)	608 (44.6)	217 (48.9)
aORs (95% CIs)	1.00 (Reference)	1.64 (1.48, 1.81)#	1.53 (1.36, 1.73)#	1.76 (1.56, 1.99)#	1.69 (1.49, 1.93)#	2.00 (1.63, 2.46)#
			1.00 (Reference)	1.15 (1.01, 1.32)*		
					1.00 (Reference)	1.18 (0.95, 1.47)
**Height at adulthood (20–25 years)**						
**1. Male**						
Smaller than standard, N (%)	4,764 (51.1)	4,809 (51.6)	2,178 (50.4)	2,631 (52.5)	1,880 (52.3)	751 (53.0)
aORs (95% CIs)	1.00 (Reference)	1.01 (0.95, 1.07)	0.96 (0.90, 1.04)	1.05 (0.98, 1.13)	1.04 (0.96, 1.13)	1.08 (0.96, 1.21)
			1.00 (Reference)	1.09 (1.00, 1.18)*		
					1.00 (Reference)	1.03 (0.91, 1.17)
**2. Female**						
Smaller than standard, N (%)	1,796 (50.8)	1,864 (52.7)	879 (50.8)	985 (54.5)	745 (54.7)	240 (54.1)
aORs (95% CIs)	1.00 (Reference)	1.08 (0.98, 1.19)	1.00 (0.89, 1.12)	1.17 (1.04, 1.32)**	1.18 (1.04, 1.34)*	1.15 (0.94, 1.41)
			1.00 (Reference)	1.17 (1.03, 1.34)*		
					1.00 (Reference)	0.98 (0.79, 1.21)

Odds ratios, 95% CIs, and *p*-value were calculated by Logistic regression with the following adjustments and covariates: age, sex, income level, Charlson comorbidity index, other drug prescriptions, and time to the health check-up.

Abbreviation: attention deficit hyperactivity disorder (ADHD), methylphenidate (MPH), body mass index (BMI), number (N), adjusted odds ratio (aOR), confidence intervals (CIs).

**p*-value<.05, ***p*-value<.01, and #*p*-value<.001.

© 2026 Song J et al. *JAMA Network Open*.

eTable 7. Association of the Prevalence of ADHD and MPH Use With BMI/Height at Adulthood, Among Children

	Children (6-11 years) at the year of ADHD					
	No ADHD	With ADHD				
		ADHD (All)	No user	User of MPH, cumulative usage [days of use]		
				User (All)	<365	365-1,460
**1. Age at end: 20-22 years**						
**BMI**						
Overweight and obesity, N (%)	2,777 (35.5)	3,672 (44.8)	1,652 (42.5)	2,020 (46.9)	1,372 (45.0)	648 (51.4)
aORs (95% CIs)	1.00 (Reference)	1.47 (1.37, 1.57)#	1.35 (1.25, 1.47)#	1.59 (1.47, 1.72)#	1.48 (1.36, 1.61)#	1.90 (1.68, 2.15)#
			1.00 (Reference)	1.17 (1.07, 1.28)#		
					1.00 (Reference)	1.29 (1.13, 1.47)#
**Height**						
Smaller than standard, N (%)	3,994 (51.0)	4,310 (52.6)	1,997 (51.3)	2,313 (53.7)	1,645 (53.9)	668 (53.0)
aORs (95% CIs)	1.00 (Reference)	1.06 (0.99, 1.13)	1.01 (0.93, 1.09)	1.12 (1.04, 1.21)**	1.13 (1.03, 1.23)**	1.10 (0.97, 1.24)
			1.00 (Reference)	1.11 (1.02, 1.21)*		
					1.00 (Reference)	0.97 (0.85, 1.11)
**2. Age at end: 23-25 years**						
**BMI**						
Overweight and obesity, N (%)	1,586 (34.3)	1,934 (45.2)	894 (44.8)	1,040 (45.5)	782 (44.7)	258 (48.0)
aORs (95% CIs)	1.00 (Reference)	1.59 (1.46, 1.74)#	1.58 (1.42, 1.76)#	1.61 (1.44, 1.78)#	1.55 (1.39, 1.74)#	1.79 (1.49, 2.16)#
			1.00 (Reference)	1.01 (0.90, 1.15)		
					1.00 (Reference)	1.15 (0.95, 1.40)
**Height**						
Smaller than standard, N (%)	2,350 (50.8)	2,149 (50.2)	970 (48.6)	1,179 (51.6)	887 (50.7)	292 (54.4)
aORs (95% CIs)	1.00 (Reference)	0.97 (0.89, 1.05)	0.91 (0.82, 1.01)	1.02 (0.92, 1.13)	0.99 (0.88, 1.10)	1.13 (0.94, 1.35)
			1.00 (Reference)	1.11 (0.99, 1.26)		
					1.00 (Reference)	1.14 (0.94, 1.39)

Odds ratios, 95% CIs, and *p*-value were calculated by Logistic regression with the following adjustments and covariates: age, sex, income level, Charlson comorbidity index, other drug prescriptions, and time to the health check-up.

Abbreviation: attention deficit hyperactivity disorder (ADHD), methylphenidate (MPH), body mass index (BMI), number (N), adjusted odds ratio (aOR), confidence intervals (CIs).

*p-value<.05, **p-value<.01, and #p-value<.001.

© 2026 Song J et al. *JAMA Network Open*.

eTable 8. Association of the Prevalence of ADHD and MPH Use With BMI/Height at Adulthood, Among Adolescents

	Adolescents (12-19 years) at the year of ADHD					
		With ADHD				
	No ADHD	ADHD (All)	No user	User of MPH, cumulative usage [days of use]		
				User (All)	<365	365-1,460
**BMI at adulthood (20–25 years)**						
Overweight and obesity, N (%)	8,525 (38.8)	9,671 (44.0)	4,892 (43.2)	4,779 (44.8)	4,009 (44.5)	770 (47.0)
aORs (95% CIs)	1.00 (Reference)	1.25 (1.20, 1.30)#	1.21 (1.15, 1.27)#	1.29 (1.23, 1.35)#	1.27 (1.20, 1.33)#	1.44 (1.30, 1.59)#
			1.00 (Reference)	1.07 (1.01, 1.13)*		
					1.00 (Reference)	1.14 (1.02, 1.26)*
**Height at adulthood (20–25 years)**						
Smaller than standard, N (%)	10,626 (48.3)	10,805 (49.2)	5,516 (48.7)	5,289 (49.6)	4,460 (49.5)	829 (50.6)
aORs (95% CIs)	1.00 (Reference)	1.03 (0.99, 1.07)	1.01 (0.96, 1.06)	1.04 (0.99, 1.09)	1.04 (0.99, 1.09)	1.08 (0.97, 1.19)
			1.00 (Reference)	1.03 (0.98, 1.09)		
					1.00 (Reference)	1.04 (0.94, 1.16)

Odds ratios, 95% CIs, and *p*-value were calculated by Logistic regression with the following adjustments and covariates: age, sex, income level, Charlson comorbidity index, other drug prescriptions, and time to the health check-up.
Abbreviation: attention deficit hyperactivity disorder (ADHD), methylphenidate (MPH), body mass index (BMI), number (N), adjusted odds ratio (aOR), confidence intervals (CIs).
**p*-value<.05, ***p*-value<.01, and #*p*-value<.001.

© 2026 Song J et al. *JAMA Network Open*.
