# Supplementary material for: ADHD and Methylphenidate Use in Prepubertal Children and BMI and Height at Adulthood
Source: JAMA Netw Open. 2026 Jan 5;9(1):e2552019. doi: 10.1001/jamanetworkopen.2025.52019 (PMC12771255; doi:10.1001/jamanetworkopen.2025.52019)
Supplement: Supplement 2. — Data Sharing Statement [file jamanetwopen-e2552019-s002.pdf]

Data Sharing Statement

Song. ADHD and Methylphenidate Use in Prepubertal Children and BMI and Height at Adulthood. *JAMA Netw Open*. Published January 05, 2026.
doi:10.1001/jamanetworkopen.2025.52019

Data
Data available: No

Additional Information
Explanation for why data not available: The original and processed data in this cohort study are only accessible to qualified researchers in permitted security facilities for a certain period since the used database was based on records of national insurance. Thus, the raw data cannot be shared openly. However, access to the code used in this study can be shared for noncommercial and academic purposes.
